# Release of 11-*cis*-retinal from cellular retinaldehyde-binding protein by acidic lipids

**Published:** 2009-04-23

**Authors:** John C. Saari, Maria Nawrot, Ronald E. Stenkamp, David C. Teller, Gregory G. Garwin

**Affiliations:** 1 Department of Ophthalmology, University of Washington, School of Medicine, Seattle, WA; 2 Department of Biochemistry, University of Washington, School of Medicine, Seattle, WA; 3 Department of Biological Structure, University of Washington, School of Medicine, Seattle, WA

## Abstract

**Purpose:**

To determine molecular mechanisms for the release of 11-*cis*-retinal from the binding pocket of cellular retinaldehyde-binding protein (CRALBP).

**Methods:**

Binding of CRALBP to lipid surfaces was assessed with a lipid-immunoblot assay. Lipids were presented to CRALBP as small unilamellar vesicles (SUVs) consisting of phosphatidylcholine (PC) plus other lipids. Release of 9-*cis*-retinal or 11-*cis*-retinal from CRALBP was measured with spectral and high performance liquid chromatography (HPLC) assays based on the protection of the protein-bound retinal carbonyl group from reaction with NH_2_OH. The electrostatic surface potential of CRALBP was calculated from a model of its structure using the program CCP4mg.

**Results:**

Incubation of CRALBP·11-*cis*-retinal with lipids absorbed on nitrocellulose revealed binding to the acidic lipids, phosphatidic acid (PA)>phosphatidylinositol 3,4,5-trisphosphate [PI(3,4,5)P_3_]>phosphatidylserine (PS)> PI(4,5)P_2_ and little or no binding to PC, phosphatidylethanolamine (PE), or PI(4)P. 11-*cis*-retinal was released during incubation of CRALBP with SUVs consisting of PC plus 50 mol% PA but not during incubation with those composed of 100 mol% PC. The efficacy of release of 9-*cis*-retinal or 11-*cis*-retinal from CRALBP by phospholipid-containing SUVs generally paralleled that of the binding of CRALBP to the lipids (PA>PS>PI>>PC). Examination of the electrostatic surface potential of the protein structure revealed a basic recess on one face of the protein, which may bind acidic lipids.

**Conclusions:**

Our results identify the first physiologic substances that release 11-*cis*-retinal from CRALBP. PA and PS are relatively minor membrane lipids that can be generated in the cytoplasmic leaflet of the plasma membrane in response to various signal transduction pathways, where they could interact with cytosolic CRALBP. The mechanism for release of retinal from CRALBP by acidic lipids remains to be determined but could involve binding of the acidic lipid in the 11-*cis*-retinal binding site or to the positive basic recess on the protein surface. These results open a new facet in our understanding of how CRALBP functions in the regeneration of visual pigments.

## Introduction

Vertebrate vision could not be maintained without a mechanism for regeneration of photoisomerized (bleached) visual pigments. Photoisomerization of 11-*cis*-retinal to all-*trans*-retinal and reduction of the retinal to all-*trans*-retinol occur in vertebrate photoreceptor cells. In adjacent retinal pigment epithelium (RPE) cells, all-*trans*-retinol is esterified with fatty acids to generate all-*trans*-retinyl esters. Enzymatic isomerization and hydrolysis of the ester bond (isomerohydrolase reaction) yields 11-*cis*-retinol and a fatty acid. 11-*cis*-retinol is oxidized to 11-*cis*-retinal by one or more *cis*-specific retinol dehydrogenases of RPE. The processes in photoreceptor and RPE cells are coupled by directed flow of 11-*cis*-retinal and all-*trans*-retinol across the interphotoreceptor matrix [1,2]. Evidence leading to the current understanding of the rod visual regeneration cycle has been presented in reviews, to which the reader is referred [3-9].

Reactions of the rod cycle appear to be well established, and the enzymes responsible for their catalysis have been identified with the exception of the dehydrogenases, where functional redundancies remain to be resolved. However, our understanding of the interactions of components and of the cell biology associated with retinoid trafficking in RPE and photoreceptor cells remains rudimentary.

There are compelling reasons to believe that cellular retinaldehyde-binding protein (CRALBP) [10] is an important component of the rod and cone visual cycles [11]. First, CRALBP has a very high affinity for 11-*cis*-retinal (K_d_ approximately 15 nM) or 11-*cis*-retinol (K_d_ approximately 53 nM) [12,13] and carries these retinoids in vivo [14]. Second, CRALBP is abundantly expressed in RPE and Müller cells [15], two cell types implicated in rod and cone visual cycles. Third, in vitro, CRALBP affected the kinetics of all reactions of the rod visual cycle that involve 11-*cis*-retinoids including those catalyzed by isomerohydrolase [16,17] and *cis*-retinol dehydrogenase (*RDH5*) [12,18]. apoCRALBP was routinely included in in vitro assays for isomerohydrolase because it stimulated the rate of the reaction so dramatically [16,17]. Fourth, regeneration of rhodopsin was delayed by roughly 15-fold in mice lacking a functional CRALBP gene (*Rlbp1^−/−^*) and analysis of rod visual cycle intermediates demonstrated a delay at the isomerohydrolase step of the visual cycle [19]. Electroretinogram (ERG) analysis also demonstrated delays in both rod and cone resensitization in these mice. Finally, humans with mutations in the CRALBP gene (*RLBP1*) displayed delays in resensitization of both cone and rod pathways [20-22].

The high affinity of CRALBP for 11-*cis*-retinoids allows the protein to accept 11-*cis*-retinol from isomerohydrolase of the rod visual cycle and facilitate its oxidation to 11-*cis*-retinal by *cis*-RDHs of RPE. However, this mechanism also leads to an apparent dead end because the same high affinity of CRALBP for 11-*cis*-retinal has to date prevented an experimental demonstration of release of retinal from the binding protein. Here we report that CRALBP bound to acidic (anionic) glycerophospholipid surfaces with specificity, and we identified a basic recess on a surface of the protein that may mediate binding. Using an assay that allowed us to measure release of 11-*cis*-retinal from CRALBP, we found that treatment of CRALBP with small unilamellar vesicles (SUVs) doped with acidic glyceropholipids, phosphatidic acid (PA) and phosphatidylserine (PS) in particular, resulted in release of 11-*cis*-retinal from the protein. These acidic glycerophospholipids are the first discovered physiologic substances that release tightly bound 11-*cis*-retinal from CRALBP. It is particularly exciting that these minor lipids are found primarily in the cytoplasmic leaflets of the plasma membrane and intracellular vesicular membranes where they could encounter CRALBP.

## Methods

### Materials

Phospholipids and their sources were as follows: dipalmitoyl phosphatidic acid (PA; Avanti Polar Lipids, Alabaster, AL); PA, from egg yolk (Sigma Aldrich, St Louis, MO); phosphatidylcholine (PC), egg yolk, bovine brain (Sigma Aldrich); phosphatidylethanolamine (PE), from egg yolk (Sigma Aldrich); phosphatidylserine (PS), bovine brain (Sigma Aldrich); PI, from bovine liver (Sigma Aldrich); phosphatidylinositol-4-phosphate (PI(4)P), from bovine brain (Calbiochem, San Diego, CA and EMD Biosciences, Gibbstown, NJ) and porcine brain (Avanti Polar Lipids); phosphatidylinositol-4,5-bisphosphate (PI(4,5)P_2_), from porcine brain (Avanti); phosphatidylinositol-3,4,5-trisphosphate (PI(3,4,5)P_3_), dioleoyl (Avanti); sphingomyelin, from bovine brain, egg yolk (Applied Science Laboratories Inc., State College, PA); gangliosides (Type III, G-2375, from bovine brain; Sigma Aldrich); sulfatides from bovine spinal cord (Applied Science Laboratories). 11-*cis*-Retinal was obtained through the generosity of the National Eye Institute (Bethesda, MD). 9-*cis*-Retinal was purchased from Sigma Aldrich. Methods for handling and analysis of retinoids have been published [23]. Anti-CRALBP was a polyclonal antibody (pAb UW55) isolated from rabbits immunized with rCRALBP.

### General

All work with light-sensitive materials (retinoids, retinoid-binding proteins) was performed in red illumination to avoid photoisomerization and photooxidation of the retinoid. Air was removed from buffers by bubbling with argon.

### Preparation of CRALBP·9-*cis*-retinal, CRALBP·11-*cis*-retinal, and rCRALBP

CRALBP was purified from bovine retina using a combination of ion exchange, lectin affinity, and hydroxylapatite chromatographies and conjugated with 9-*cis*- or 11-*cis*-retinal as described previously [24]. rCRALBP was expressed in *E. coli* as a His-tagged protein, purified via a Ni-affinity column, and conjugated with 9-*cis*-retinal.

### Lipid-immunoblot assay

The lipid-immunoblot assay we employed is a variation on that described for determining the specificity of pleckstrin homology domains for phosphoinositides [25]. Various glycerophospholipids were dissolved in CHCl_3_ and small volumes (<5 μl, 5–25 nmol) were applied to nitrocellulose membranes (Hybond ECL, GE Healthcare, Little Chalfont, Buckinghamshire, UK) with drying. Afterwards, the membrane was incubated with 10 μg/ml CRALBP in Tris-buffered saline Tween (TBST, 10 mM Tris, pH 8, 0.15 M NaCl, 0.05% Tween-20). The membrane was then washed with TBST containing 2% milk proteins (from dried non-fat milk, Lucerne Dairies, Seattle, WA) and incubated sequentially with anti-CRALBP (UW55 pAb), anti-rabbit IgG coupled to alkaline phosphatase, and a luminescent alkaline phosphatase substrate (Lumi-Phos WB Pierce, Thermo Fisher Scientific, Pittsburgh, PA). Milk protein was included in the antibody solutions to reduce the background. Blots were incubated with X-ray film for chemiluminescent detection. Controls included application of solvent only, omission of CRALBP, and substitution of other proteins for CRALBP.

### Assays for release of retinal from CRALBP

CRALBP has a high affinity for 9-*cis*-retinal and 11-*cis*-retinal [12,13]. In addition, the carbonyl of either retinal bound to CRALBP is sequestered from reaction with water-soluble carbonyl reagents [18,26]. We exploited these properties to develop assays for the release of retinal from CRALBP. Commercially available 9-*cis*-retinal was used as a surrogate for 11-*cis*-retinal in many of these studies, but all results have been repeated with 11-*cis*-retinal. The spectral assay was based on differences in the spectral properties of 9-*cis*-retinal, 9-*cis*-retinal bound to CRALBP, and 9-*cis*-retinal oxime. The high performance liquid chromatography (HPLC) assay involved extraction of bound retinoids after incubation and identification and quantification of *cis*-retinals and *cis*-retinal oximes. These assays were performed in dim red illumination to avoid photoisomerization of bound retinal.

### Spectral assay for release of retinal from CRALBP

CRALBP·9-*cis*-retinal or CRALBP·11-*cis*-retinal (6 or 12 μM) in 20 mM MOPS, pH 7.2, was incubated at room temperature in the dark with 2 mM NH_2_OH (freshly neutralized NH_2_OH·HCl). SUVs of various compositions were added (90 μM total phospholipid concentration) and UV/Vis spectra were recorded at intervals. The absorbances of 9-*cis*-retinal and 11-*cis*-retinal are shifted from approximately 365 nm (hexane) to 410 nm and 425 nm, respectively, when bound to CRALBP. Retinal oximes absorb at 355 nm. Thus, the release of either retinal from CRALBP for reaction with NH_2_OH was registered as a decrease in absorbance at 410 or 425 nm and an increase in absorbance at approximately 355 nm.

### HPLC assay for release of retinal from CRALBP

Release of 9-*cis*-retinal or 11-*cis*-retinal from CRALBP was also monitored by extraction of the retinoids and quantification by HPLC analysis. CRALBP·9-*cis*-retinal (12 μM) in 20 mM MOPS, pH 7.2, was incubated with 2 mM NH_2_OH at room temperature. At intervals, portions were removed and incubated with pyridoxal 5′-phosphate to inactivate NH_2_OH. After 10 min, ethanol was added to 50% (v/v), and retinoids were extracted into hexane by following published procedures. Retinoids were resolved and quantified with a Phenomenex Luna 3 silica (2) column (150×4.6 mm, 3 μm) and isocratic elution with hexane: ethyl acetate: octanol (90/10/1, v/v). Methods used for extraction and retinoid analysis have been previously described [23]. After incubation of CRALBP·9-*cis*-retinal, an additional peak appeared in the chromatogram, which migrated close to 9-*cis*-retinal. We are uncertain of the identity of this component. Its spectrum, obtained on the fly, was indistinguishable from that of 9-*cis*-retinal. No additional oximes appeared upon incubation with NH_2_OH.

### Preparation of SUVs

The general method used was that of Barenholz et al. [27] as described by Fang et al. [28]. Phospholipids (50 mg) were dissolved in CHCl_3_, dried with flowing argon, resuspended in 3 ml of buffer, which contained 50 mM MOPS, pH 7.2, 100 mM KCl, 1 mM EGTA, and 1 mM MgCl_2_. The mixture was then sonicated on ice with a micro sonicator probe operated at 70W (Sonic Dismembrator, Model 100, Fischer Scientific) until the solution was clear. Next, 0.1 mM diethylenetriaminepentaacetic acid (DTPA) was added to inhibit oxidative decomposition of lipids. SUVs were recovered in the supernatant after centrifuging at 60,000 ×g at 25 °C for 2 h.

### CRALBP model structure

Coordinates for a molecular model of CRALBP were analyzed with molecular visualization software RasMol [29], Chime, MIFit [30], and XtalView [31] to examine the distribution of basic residues on the surface of the protein. Electrostatic surface potential was calculated and viewed using CCP4mg [32].

## Results

### CRALBP bound to a select set of lipids

**Figure 1:**
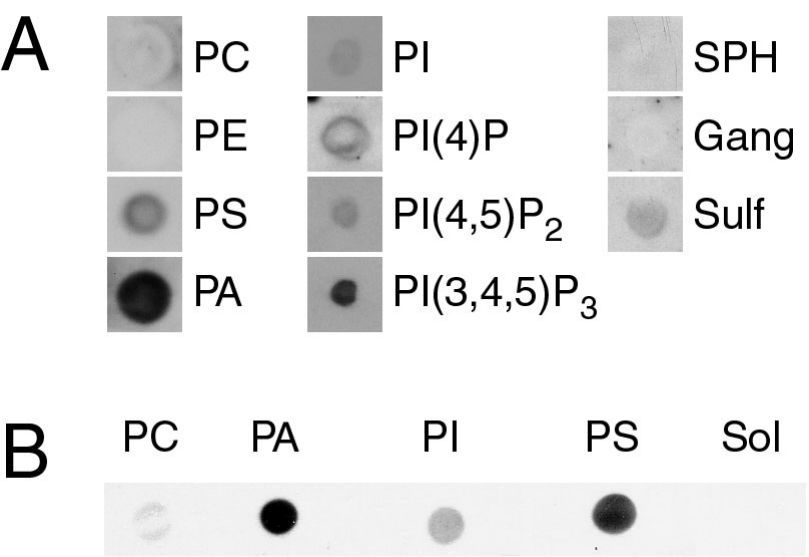
Lipid-immunoblot assay. **A:** Several lipids were surveyed. Lipids were dissolved in CHCl_3_ and applied to nitrocellulose membranes in small spots (25 nmol/spot). After the membrane was appropriately blocked and washed, it was probed with bovine cellular retinaldehyde-binding protein (CRALBP) and anti-CRALBP as described in Methods. CRALBP bound tightly to surfaces coated with the acidic glycerophospholipids, phosphatidic acid (PA), phosphatidylinositol-3,4,5-tris-phosphate [PI(3,4,5)P_3_], phosphatidylserine (PS), phosphatidyl-4-phosphate (PI(4)P), and more weakly to surfaces coated with the other acidic lipids phosphatidylinositol (PI), phosphatidylinositol-4,5-bisphosphate (PI(4,5)P_2_), and a mixture of sulfatides (Sulf). No binding was apparent with phosphatidylcholine (PC), phosphatidylethanolamine (PE), sphingomyelin (SPH), or a mixture of gangliosides (Gang). Each of the three columns in the figure was derived from a separate immunoblot. The size of the lipid spots was variable due to components in the lipid solutions that affected wetting of the nitrocellulose. The results shown are representative of at least three independent analyses of each lipid. **B:** Active lipids were compared on the same blot. The lipids indicated below the blot were applied to nitrocellulose in small spots (25 nmol/spot) and probed with bovine CRALBP as described for **A**. The order of staining intensity (PA>PS>PI>PC) was observed in at least three independent analyses. Solvent is abbreviated Sol.

We were led to examine whether CRALBP could bind to lipids because the ligand-binding domain of the protein is derived from that of yeast SEC14, a protein that mediates exchange of PI and PC [33]. Various membrane lipids were dissolved in CHCl_3_ and applied to nitrocellulose membranes in small spots (5–25 nmol lipid/spot). After nonspecific binding sites were blocked, the membrane was probed with CRALBP isolated from bovine retina. CRALBP that was retained on the membrane was detected by anti-CRALBP, alkaline phosphatase coupled to anti-IgG, and a chemiluminescent substrate. Figure 1A illustrates the results obtained with some lipids found in biologic membranes. In general, CRALBP bound to acidic (anionic) lipids but even within this class there was considerable variation in specificity. PA, a dianionic glycerophospholipid, and PI(3,4,5)P_3_, a polyanionic glycerophospholipid, bound CRALBP avidly, as judged by the intensity of staining with anti-CRALBP. The intensity of staining with PS, PI(4)P, and PI(4,5)P_2_ was less than that observed with PA and PI(3,4,5)P_3_ but well above background. CRALBP did not bind to neutral (zwitterionic) lipids such as PC, PE, and sphingomyelin or to a mixture of gangliosides. A mixture of sulfatides showed weak binding. A comparison of the binding of CRALBP to several lipids on the same blot is shown in Figure 1B. Based on the intensity of staining, the binding efficiency was PA>PS>PI>PC. CRALBP bound to both PA isolated from natural sources and to synthetic dipalmitoylPA, suggesting that the fatty acids esterified to glycerol were not important determinants of binding (results not shown). Apo-CRALBP and CRALBP·11-*cis*-retinol both bound to PA and PS surfaces suggesting that bound retinoid was not required for binding (results not shown). Further studies were pursued with PA because this lipid has been demonstrated to control protein binding to membranes [34-37] and because it is relatively abundant in biologic membranes [38].

### Validation of assays for release of retinal from CRALBP

**Figure 2:**
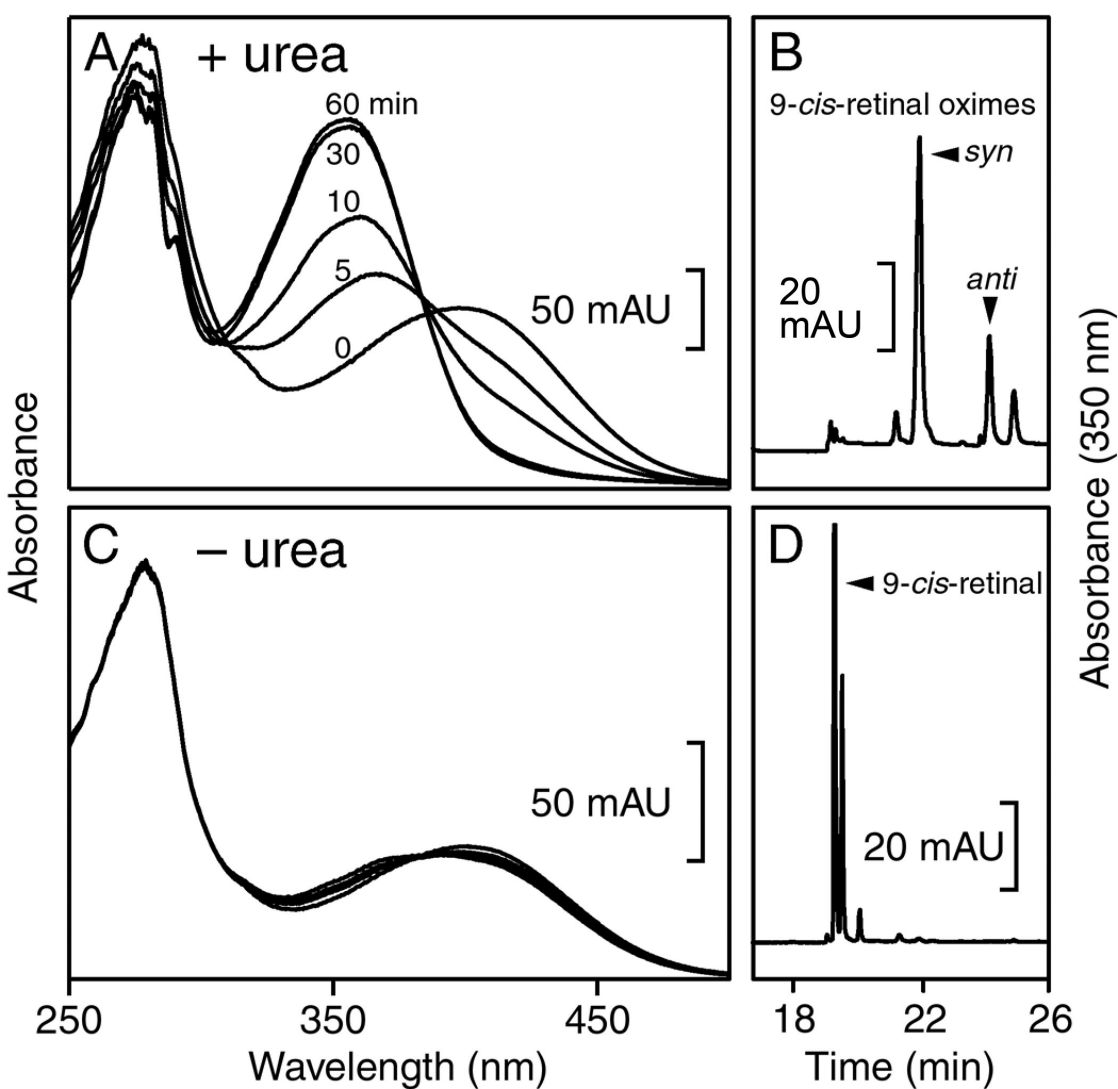
Validation of assays for release of *cis*-retinal from CRALBP. Cellular retinaldehyde-binding protein (CRALBP) ·9-*cis*-retinal (12 μM) was incubated in the dark with 2 mM NH_2_OH in the presence or absence of 4 M urea. Incubation of the protein with 4 M urea resulted in a progressive loss of absorbance at 410 nm and an increase in absorbance at 355 nm (**A**). The retinoid was recovered by extraction and high performance liquid chromatography (HPLC) analysis as 9-*cis*-retinal oxime (**B**). During 60 min of incubation without urea, there was minimal shift of the ligand UV absorption maximum (**C**). The retinoid was recovered by extraction and HPLC analysis as 9-*cis*-retinal and an unidentified retinoid, possibly a di-*cis*-retinal (**D**). The results shown in the panels are representative of at least six independent repeats of the experiment.

Lack of release of the chromophore during incubation of CRALBP·9-*cis*-retinal in the presence of NH_2_OH at neutral pH and room temperature emphasized that the aldehyde group of the retinoid was remarkably protected from the carbonyl reagent. When 4 M urea was included in the incubation mixture, the absorption maximum of the bound retinal shifted progressively from approximately 400 nm to approximately 355 nm and after 3 h of incubation the retinoid was nearly completely recovered as 9-*cis*-retinal oxime (Figure 2A,B). In the absence of urea, the absorption spectrum of the bound retinoid was little affected during 3 h of incubation and >95% of the chromophore was recovered as 9-*cis*-retinal and not as the oxime (Figure 2C,D). Incubation of CRALBP·9-*cis*-retinal or 11-*cis*-retinal with NH_2_OH with various additives such as glycerophospholipids provided the basis for the release assay to be described in subsequent experiments.

### PA released 9-*cis*-retinal from native bovine CRALBP

**Figure 3:**
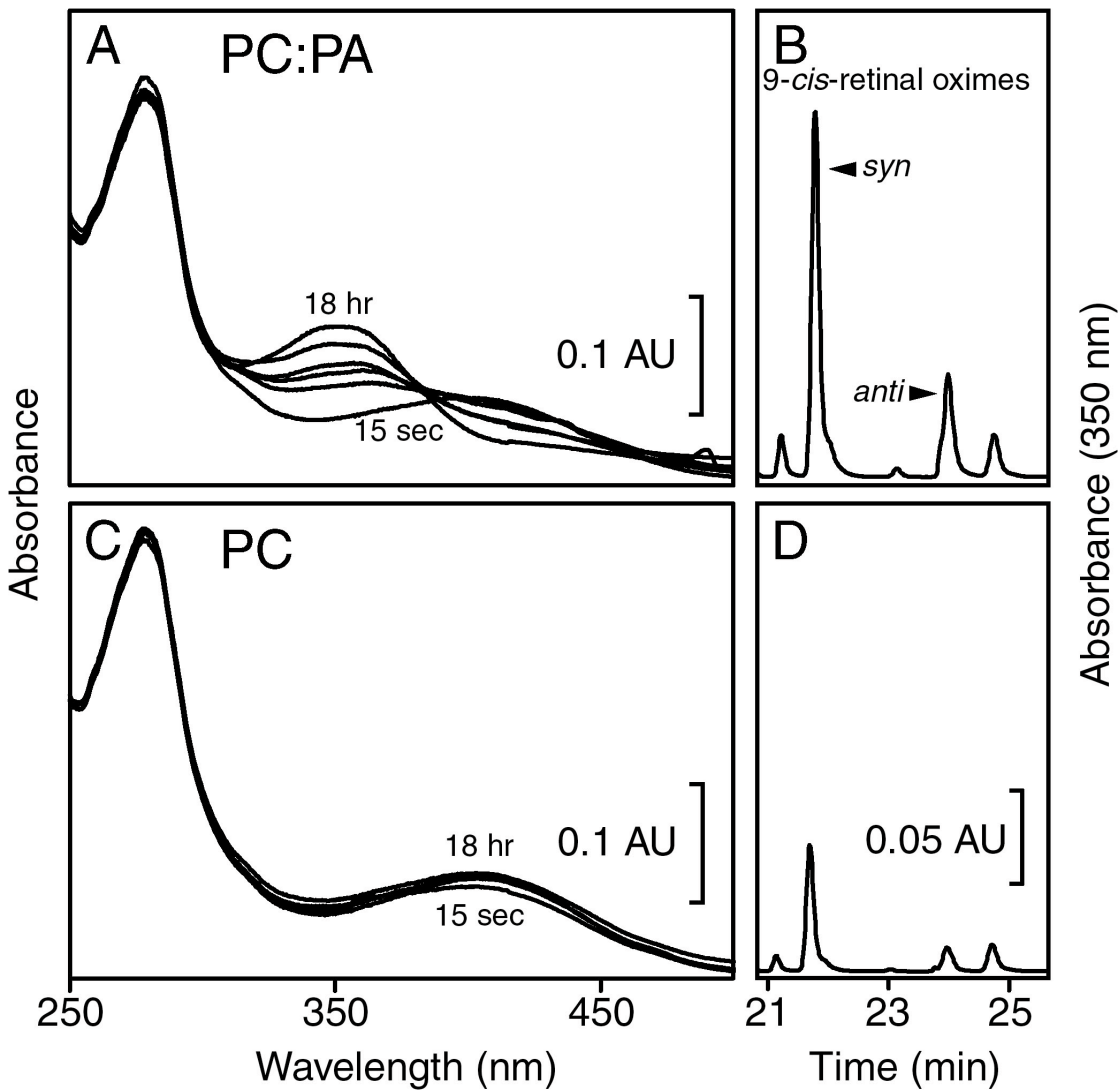
9-*cis*-Retinal was released from CRALBP by acidic glycerophospholipids. Cellular retinaldehyde-binding protein (CRALBP)·9-*cis*-retinal was incubated in the dark with NH_2_OH and small unilamellar vesicles (SUVs). Release of 9-*cis*-retinal was followed using the spectral assay (**A** and **C**) and the HPLC assay (**B** and **D**). Incubation of CRALBP·9-*cis*-retinal with SUVs composed of 50 mol% phosphatidylcholine (PC) and 50 mol% phosphatidic acid (PA) produced spectral changes over 18 h consistent with release of 9-*cis*-retinal for reaction with NH_2_OH (**A**), and high performance liquid chromatography (HPLC) analysis of an 18 h sample demonstrated recovery of 9-*cis*-retinal oximes (**B**). Incubation with SUVs composed of 100 mol% PC had little effect on the absorption spectrum of 9-*cis*-retinal bound to CRALBP (**C**), and minimal amounts of 9-*cis*-retinal oximes were recovered (**D**). The amount of 9-*cis*-retinal released in **A** was approximately 60% of the total bound to the protein. In **B** and **D**, only the oxime regions of the chromatograms are shown. The data shown in the panels are representative of at least five independent repeats of the experiment.

CRALBP·9-*cis*-retinal was incubated with NH_2_OH and SUVs using the conditions of the release assays. Incubation with SUVs composed of 50 mol% PC and 50 mol% PA produced spectral changes consistent with release of 9-*cis*-retinal for reaction with NH_2_OH (Figure 3A), and HPLC analysis verified increased recovery of 9-*cis*-retinal oximes after 18 h (Figure 3B). The amount of retinal released in this experiment was roughly 60% of that bound to CRALBP. SUVs composed solely of PC had little effect on the absorption spectrum of 9-*cis*-retinal bound to CRALBP (Figure 3C), and little 9-*cis*-retinal oxime was recovered after 18 h of incubation (Figure 3D). The release of 9-*cis*-retinal was comparable to that observed during incubation of the binding protein in buffer for 18 h (result not shown) and is likely due to thermal instability of the protein.

### Specificity of release of 11-cis-retinal and 9-cis-retinal from CRALBP

**Figure 4:**
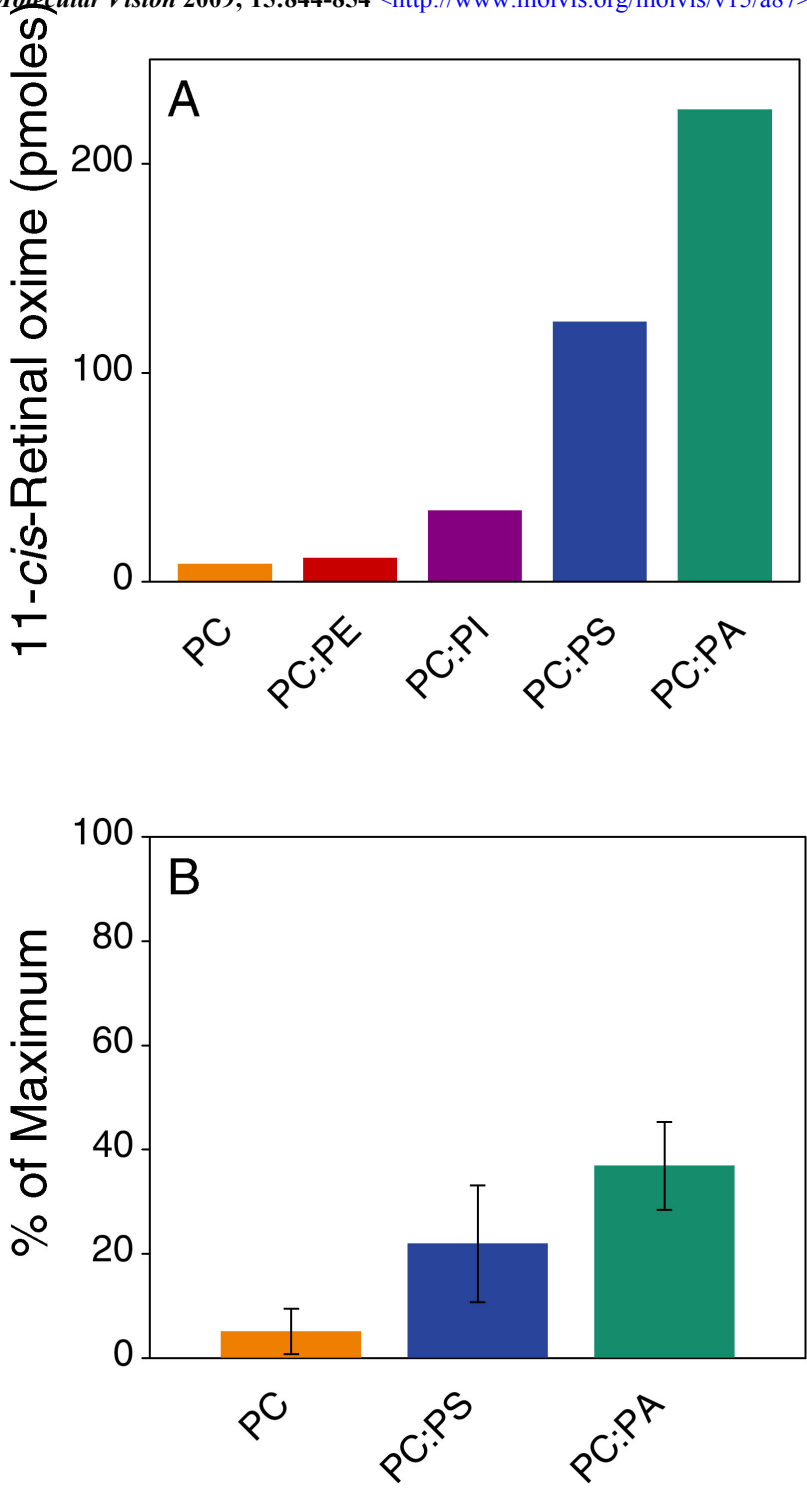
Release of 11-*cis*- and 9-*cis*-retinal from CRALBP. **A:** Cellular retinaldehyde-binding protein (CRALBP)·11-*cis*-retinal (6 μM) was incubated in the dark with 2 mM NH_2_OH and small unilamellar vesicles (SUVs). The total phospholipid concentration was 90 μM, and SUVs were composed of 100 mol% phosphatidylcholine (PC) or 50 mol% PC and 50 mol% other lipids, as indicated. After 16 h at room temperature, retinoids were extracted and quantified by high performance liquid chromatography. The amount of 11-*cis*-retinal oximes (syn+anti) reflected the release of 11-*cis*-retinal for reaction with NH_2_OH during the incubation. The amount of 11-*cis*-retinal released from by PC: phosphatidic acid (PA) was approximately 75% of the total bound to CRALBP. The results shown are from a single experiment. **B:** CRALBP·9-*cis*-retinal (6 μM) was incubated in the dark with NH_2_OH and SUVs as described. Spectra were obtained before and 3 h after addition of the SUVs. The results are shown as the % of the maximum spectral change resulting from incubation with 4 M urea. Error bars shown are standard deviations from the means (n=3). The abbreviations used are: phosphatidic acid (PA); phosphatidylcholine (PC); phosphatidylethanolamine (PE); phosphatidylinositol (PI); phosphatidylserine (PS).

To explore the specificity of release, we incubated 6 μM CRALBP·11-*cis*-retinal with 2 mM NH_2_OH and SUVs of varying compositions (Figure 4A) for a total phospholipid concentration of 90 μM. After 16 h at room temperature, retinoids were extracted and quantified by HPLC. The amount of 11-*cis*-retinal oximes (syn + anti) was indicative of the release of 11-*cis*-retinal for reaction with NH_2_OH during the incubation. The order of release activity from this experiment was the same as that derived from the lipid-immunoblot assay (PA>PS>>PI~PC; Figure 2). As with the binding assay, PC and PE showed minimal activity. We also employed the spectral assay to determine the relative order of release resulting from incubation of CRALBP·9-*cis*-retinal with SUV for 3 h (Figure 4B). The same order of release was observed with a more limited set of lipids (PA>PS>PC).

### Concentration dependence of release

**Figure 5:**
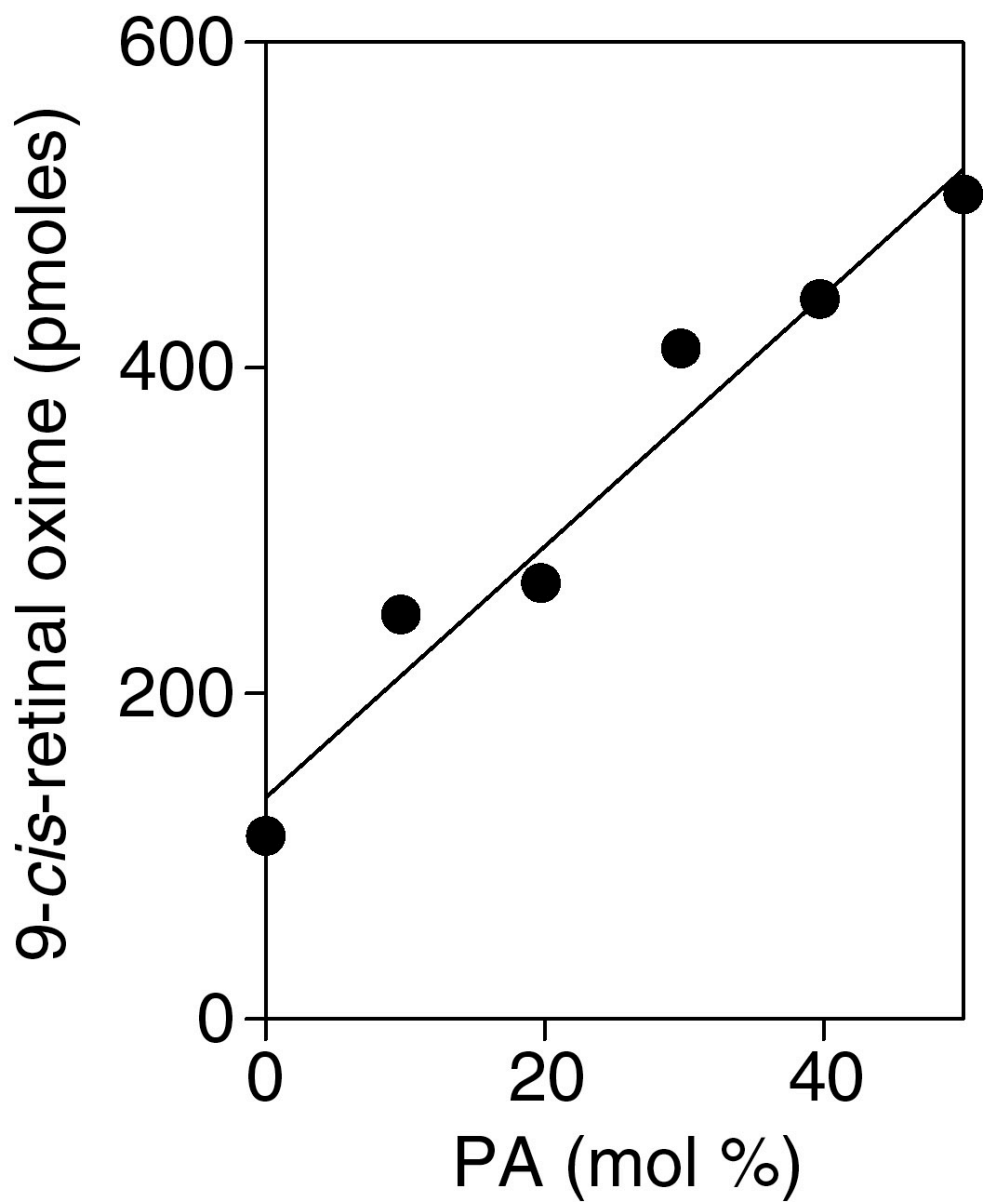
Release of retinal as a function of PA content of SUVs. CRALBP×9-*cis*-retinal (6 μM) was incubated with NH_2_OH and SUVs composed of PC and varying amounts of PA (total glycerophospholipid 90 μM). The mole % of PA in the SUVs in indicated on the abscissa. After incubation for 16 h at room temperature in the dark, retinoids were extracted and quantified by HPLC analysis. The results shown are representative of two independent repeats of the experiment.

CRALBP·9-*cis*-retinal (6 μM) was incubated under release assay conditions with SUVs composed of PC and various mol% of PA for a total concentration of 90 μM lipid. The results shown in Figure 5 indicate that the extent of release was proportional to the mol% of PA in the SUV over the range of compositions examined.

### Model of CRALBP structure shows a cluster of basic amino acid side chains

**Figure 6:**
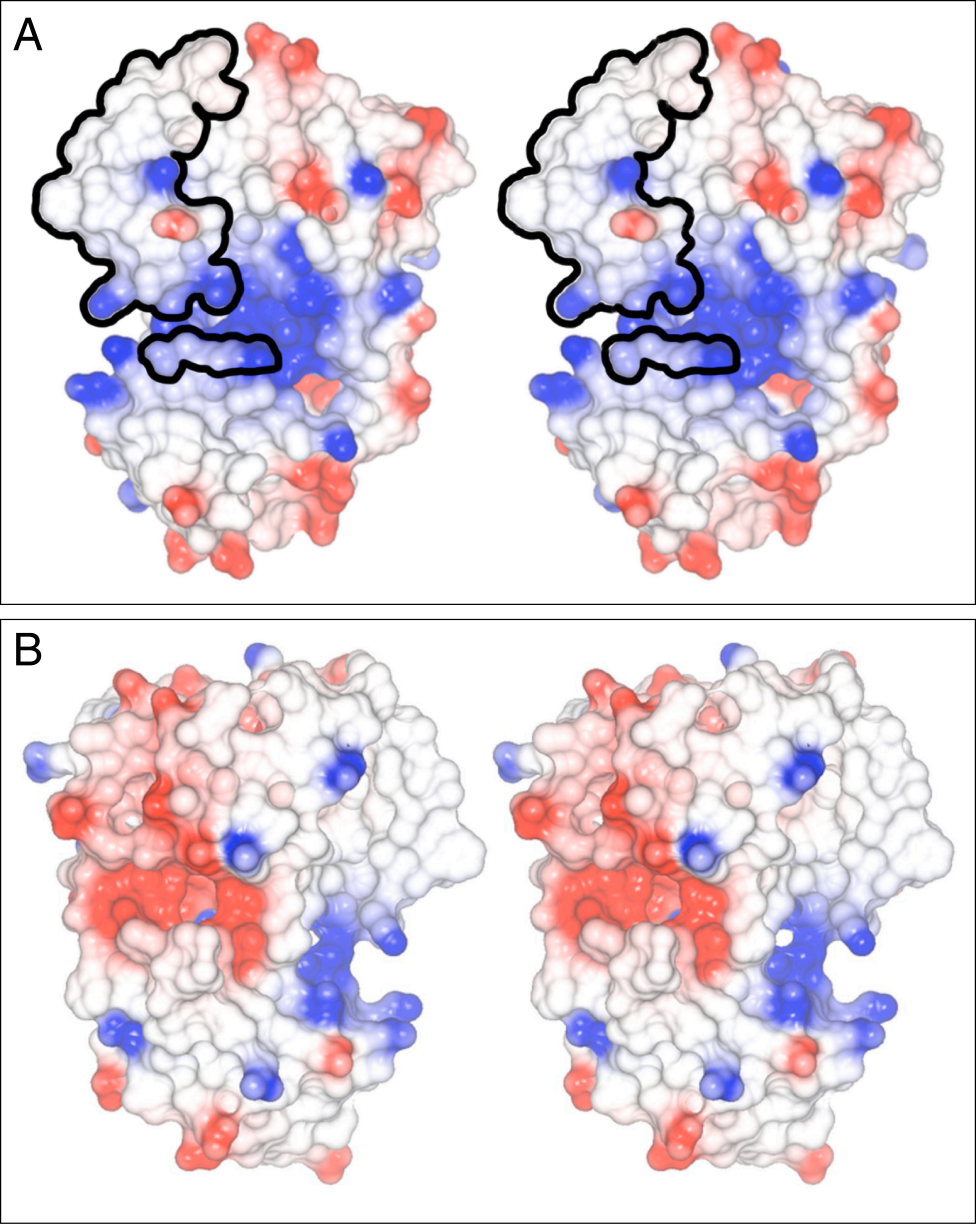
Electrostatic surface potential of CRALBP. The structural model of cellular retinaldehyde-binding protein (CRALBP; PDB entry 1XGH) is that of Liu et al. [40]. Surface regions in red have negative electrostatic potential and are acidic. Those in white have neutral electrostatic potential and those in blue have positive electrostatic potential and are basic. **A:** The two frames form a stereo pair of the basic face of CRALBP. The basic recess (blue) between the upper and lower lobes of CRALBP is approximately 10 Å in diameter. An Arg-Ala-Arg (RAR) sequence, conserved throughout the CRAL_TRIO protein family, is shown (small patch of circled residues). The retinoid-binding site is covered by the lipid exchange loop (large patch of circled residues). The basic recess may mediate interactions of CRALBP with specific acidic lipids. **B:** The two images form a stereopair of the acidic face of the protein. Note the (negative) acidic patch (red).

The crystal structures of four members of the CRAL_TRIO family of proteins were used to construct structural models of CRALBP [38,39]. The electrostatic surface potentials of two faces of CRALBP were calculated and are shown in Figure 6. On one face of the model (Figure 6A, blue residues), a basic recessed area was immediately obvious between upper and lower lobes of the protein. Basic residues lining this recess were K103, K152, K258, K260, R97, R100, R102, R108, R150, R152, and H235. The diameter of the recess was approximately 10 Å. A lipid-exchange loop (CRALBP residues 141–152, Figure 6A), which has been suggested to regulate the release of bound ligand from the protein [40-42], is outlined in the upper left quadrant of Figure 6A. Three amino acids conserved in the CRAL_TRIO family (RAR, CRALBP residues 100–102, lower outlined cluster) are present in proximity to the rim of the basic recess. These residues have been suggested to play a role in regulation of access to the ligand-binding cavity. An acidic patch located below the ligand-exchange helix (Figure 6B, red) characterized another face of the protein.

## Discussion

Previous in vitro studies provided mechanisms for loading apo-CRALBP with 11-*cis*-retinol [16,17] and for oxidation of CRALBP·11-*cis*-retinol to CRALBP·11-*cis*-retinal [18,26]. If 11-*cis*-retinoids flow through the binding site of CRALBP, as these findings imply, a subsequent step must involve release of the 11-*cis*-retinal from the binding protein to another component, to the exterior of the RPE cell, or possibly to the interior of a recycling vesicle. However, incubation of CRALBP·11-*cis*-retinal with numerous putative acceptors, cofactors, as well as RPE tissue fractions did not release retinal from the protein. For instance, 11-*cis*-retinal was not released from CRALBP during incubation with RPE microsomes±3 mM ATP, RPE microsomes±0.1 mM reduced glutathione/1 mM oxidized glutathione, RPE microsomes±interphotoreceptor retinoid-binding protein (IRBP; equimolar with CRALBP), proteases (matrix metalloproteinases 2 or 9), and anti-CRALBP (threefold molar excess; Garwin and Saari, results not shown). CRALBP is an eponymous member of the CRAL_TRIO family of proteins whose lipid-binding domain was derived from that of the yeast SEC14 protein [32]. SEC14 mediates transfer of PC and PI between Golgi and plasma membranes and is necessary for secretion in yeast. Thus, the evolutionary origins of CRALBP led us to consider whether glycerophospholipids might be capable of releasing 11-*cis*-retinal from the protein.

The lipid-immunoblot assay employed in this study clearly demonstrated that CRALBP bound to a select set of lipid surfaces. Specificity was evident at two levels. First, of the lipids tested, CRALBP only bound to acidic lipids. This eliminated the possibility that binding was an artifact caused by denaturation of CRALBP on the lipid surface. Second, CRALBP bound to some acidic lipids more avidly than to others with an apparent order of binding affinity PA > PI(3,4,5)P_3_ > PS. This suggested that binding was mediated by spatial recognition and not simply by the charge of the lipid. It is all the more remarkable that CRALBP binds to acidic surfaces because its overall charge is acidic at neutral pH (pI ~5).

Incubation of CRALBP·11-*cis*-retinal with SUVs of PC plus other glycerophospholipids demonstrated specificity for release of 11-*cis*-retinal. As observed in the binding assay, PA was the most effective lipid of those we tested in releasing 11-*cis*-retinal from CRALBP. The order of efficacy for release matched the order of effectiveness of binding to lipid surfaces, suggesting that the release mechanism may be related to binding of CRALBP to anionic lipids in the SUV. However, other mechanisms are possible as discussed in this section, including direct binding of the lipid in the 11-*cis*-retinal–binding site. It should also be noted that the lipid-blot assay we employed is qualitative and many parameters remain to be established. For instance, retention of the lipids on the nitrocellulose membrane during washings and incubations could vary from lipid to lipid and the state of the lipid adsorbed to the membrane has not been characterized. Nonetheless, the results were useful in identifying lipids that were active in releasing 11-*cis*-retinal from CRALBP.

The acidic glycerophospholipids we identified here are the first physiologic compounds found that affected the affinity of CRALBP for 11-*cis*-retinal. Which one, if any, of the acidic glycerophospholipids is relevant in RPE cells remains to be determined. Phosphoinositides are important in mammalian signaling because their synthesis can be stimulated and because they can be recognized by various protein motifs [38]. However, in most membranes they are minor lipids (<0.1 mol%) and would appear poorly suited for stoichiometric processes. For instance, the level of PI(3,4,5)P_3_, the most active phosphoinositide in our study, is 0.005 to 0.0005 that of PI [38,43]. In contrast, PS and PA are the main acidic phospholipids of mammalian cells. Concentrations of PS in the inner leaflet of the plasma membrane can reach 25 mol% [38]. PA, in particular, has recently attracted a great deal of attention because of its unique properties [34,35,44]. Localization in the cytoplasmic leaflet of the plasma membrane [38] and generation in response to activation of various signaling pathways [37,45] provide rationales for its ability to control the activity or subcellular localization of various proteins [46,47].

Models of CRALBP structure show a cluster of basic residues on a face of the protein adjacent to the “ligand exchange helix.” Binding of the protein to acidic lipid surfaces may occur via this patch, a hypothesis that can be tested through site-generated mutagenesis. It is interesting to note that several disease-causing missense mutations involve basic residues (R150Q, R150W, R233W, R234W/R103W compound heterozygote) [48]; however, of these only R150 appears to contribute to the basic recess. We do not understand the significance of the acidic patch on another face of the protein (Figure 6B). However, its presence emphasizes the nonrandom distribution of acidic and basic residues and further suggests that CRALBP might interact with other proteins or substances via this acidic patch.

Two mechanisms for release of 11-*cis*-retinal appear likely. 1) Direct binding of the acidic glycerophopholipid in the 11-*cis*-retinal binding pocket should be considered because the ligand-binding domain of CRALBP is derived from the glycerophospholipid-binding domain of SEC14. It is possible that CRALBP has retained some of the gycerophospholipid-binding properties of its ancestor. In this mechanism, dissociation of 11-*cis*-retinal from CRALBP would be followed by binding of the acidic lipid. However, the mechanism implies that the acidic lipid has an affinity for the protein equal to or greater than that of 11-*cis*-retinal and CRALBP, purified from RPE homogenates in the absence of exogenous retinoid, was nearly completely saturated with 11-*cis*-retinal [14] even though the homogenates contained abundant amounts of glycerophospholipids. Determination of the amount of radioactivity associated with CRALBP after incubation with PC-SUVs doped with [^14^C] PA should resolve this issue. 2) It is also possible that the ligand-binding domain of CRALBP is perturbed when CRALBP binds to acidic lipids in SUVs experimentally or the plasma membrane in vivo, resulting in diminished affinity and release of the ligand. The basic recess of CRALBP appears immediately below the ligand-binding domain of the protein (Figure 6A). Neutralization of the basic charges on the protein by acidic lipids could be followed by a structural rearrangement, which would decrease the affinity of CRALBP for 11-*cis*-retinal. A first step in analyzing this mechanism would be to determine whether CRALBP binds to SUVs or other suitable membrane surrogates doped with acidic glycerophospholipids.

The role of the SUVs in these release experiments has not yet been established. They could mimic a membrane surface and provide a docking site for CRALBP via an acidic lipid as discussed in the previous section. Alternatively, the SUVs could simply provide a vehicle for solubilization of PA or other acidic lipids, which could then bind to CRALBP in the basic recess or in the retinoid-binding site. The average number of glycerophospholipids/SUV is approximately 2,300 [49]. Thus, the SUV concentration in our experiments (90 μM ÷ 2300) is 39 nM—154 fold lower than the concentration of CRALBP·9-*cis*-retinal. This rules out a mechanism involving stoichiometric binding of CRALBP to an SUV unless the complex dissociates after release of the retinal. However, the results appear consistent with the SUVs simply solubilizing PA or another acidic lipid and delivering the lipid to CRALBP. Even at 10 mol%, the lowest mol% of PA in the SUVs, the input concentration of PA is greater than the concentration of CRALBP·9-*cis*-retinal. Results from the additional experimental approaches to the mechanism are needed to decide the issue.

It is clear from these studies that PA and other acidic lipids release 11-*cis*-retinal from CRALBP. However, there are many other naturally occurring acidic substances that could also be active, including fatty acids, acidic peptides, or acidic motifs/domains of other proteins. Further experimentation is necessary to determine the potential roles of these substances.

**Figure 7:**
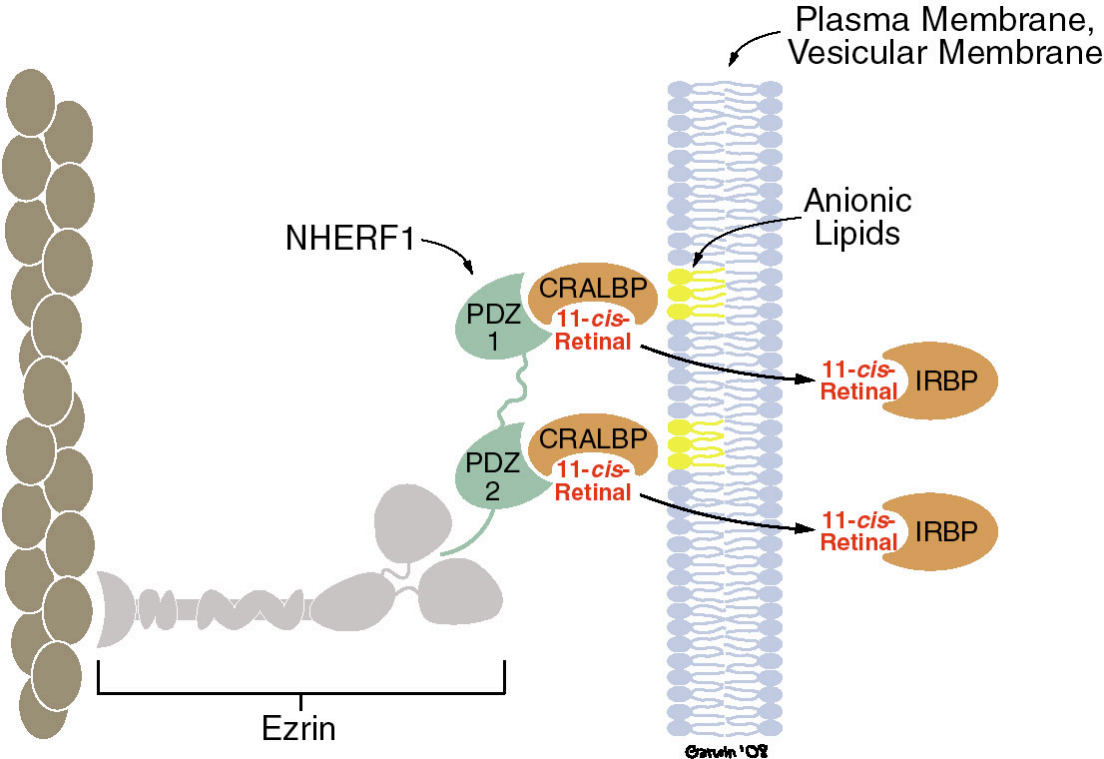
Hypothesis for release of retinal by glycerophospholipids. Cellular retinaldehyde-binding protein (CRALBP) binds to sodium hydrogen exchanger regulatory factor type 1 (NHERF1) via interactions with post synaptic density 95-discs large-zonula occludens (PDZ1), and perhaps PDZ2 [9,51,52]. NHERF1 binds to ezrin, an actin-binding scaffold protein. NHERF1, ezrin, actin, and CRALBP are found in retinal pigment epithelium apical processes. We suggest that binding of CRALBP to this or a similar complex restricts the diffusion of CRALBP in the vicinity of acidic glycerophospholipids of the inner leaflet of the plasma membrane or perhaps a vesicular membrane. Image modified from Nawrot et al. [52].

The rate of release of 11-*cis*-retinal from CRALBP in the experiments described here appears to be too slow for the process to be a factor in a physiologic mechanism. In addition, the 50 mol% PA used in most of the experiments here is much higher than reported in naturally occurring membranes. However, it is possible that mechanisms exist in vivo to accelerate the rate of release with a lower concentration of PA. For instance, we noted that 9-*cis*-retinal was released much more rapidly by PA from rCRALBP than from the native protein (t_1/2_ approximately 30 min compared to several hours, respectively; results not shown). rCRALBP has a sequence of nine histidines at its N-terminus [50], which could bind to PA-doped SUVs and increase the concentration of PA in the vicinity of the basic recess or ligand-binding domain. Previous studies have established that CRALBP binds to the post synaptic density95-discs large-zonula occludens (PDZ)-domain protein sodium-hydrogen exchanger regulatory factor type 1 (NHERF1) via interactions with PDZ1 and perhaps PDZ2 [51,52]. NHERF1 in turn, can bind to ezrin, an actin-binding scaffold protein [53]. All of these components are found in RPE apical processes [50]. Formation of a complex with CRALBP, NHERF1, ezrin, and actin in RPE apical processes could increase the concentration of the binding protein in the vicinity of the inner leaflet of the plasma membrane and thus increase the rate of release of 11-*cis*-retinal (Figure 7). This hypothesis could be approached experimentally by determining whether PA released 11-*cis*-retinal from CRALBP in isolated complexes with NHERF1, or NHERF1 and ezrin [52].

In summary, we have demonstrated that CRALBP bound strongly to PA and more weakly to a restricted set of acidic lipids. PA, presented as a component of an SUV, released 11-*cis*- or 9-*cis*-retinal from CRALBP more effectively than any other of the major acidic lipids tested and in general, the order of efficacy for release matched that for binding. A recess lined with basic (positively charged) residues was found on a surface of a model of CRALBP and could mediate binding of acidic (negatively charged) lipids and their ability to release 11-*cis*-retinal. Acidic glycerophospholipids are the first naturally occurring substances discovered that release 11-*cis*-retinal from CRALBP.
